# Decellularized squid mantle scaffolds as tissue‐engineered corneal stroma for promoting corneal regeneration

**DOI:** 10.1002/btm2.10531

**Published:** 2023-05-09

**Authors:** Honghua Kang, Yi Han, Mengyi Jin, Lan Zheng, Zhen Liu, Yuhua Xue, Zuguo Liu, Cheng Li

**Affiliations:** ^1^ Eye Institute & Affiliated Xiamen Eye Center, School of Medicine Xiamen University Xiamen China; ^2^ Fujian Provincial Key Laboratory of Ophthalmology and Visual Science, School of Medicine Xiamen University Xiamen China; ^3^ School of Pharmaceutical Sciences Xiamen University Xiamen China; ^4^ Department of Ophthalmology the First Affiliated Hospital of University of South China Hengyang Hunan China

**Keywords:** corneal stromal equivalents, corneal tissue engineering, decellularized squid mantle, marine biomaterials, tissue clearing technology

## Abstract

Corneal blindness is a worldwide major cause of vision loss, and corneal transplantation remains to be the most effective way to restore the vision. However, often there is a shortage of the donor corneas for transplantation. Therefore, it is urgent to develop a novel tissue‐engineered corneal substitute. The present study envisaged the development of a novel and efficient method to prepare the corneal stromal equivalent from the marine biomaterials‐squid. A chemical method was employed to decellularize the squid mantle scaffold to create a cell‐free tissue substitute using 0.5% sodium dodecyl sulfate (SDS) solution. Subsequently, a novel clearing method, namely clear, unobstructed brain imaging cocktails (CUBIC) method was used to transparent it. Decellularized squid mantle scaffold (DSMS) has high decellularization efficiency, is rich in essential amino acids, and maintains the regular fiber alignment. In vitro experiments showed that the soaking solution of DSMS was non‐toxic to human corneal epithelium cells. DSMS exhibited a good biocompatibility in the rat muscle by undergoing a complete degradation, and promoted the growth of the muscle. In addition, the DSMS showed a good compatibility with the corneal stroma in the rabbit inter‐corneal implantation model, and promoted the regeneration of the corneal stroma without any evident rejection. Our results indicate that the squid mantle can be a potential new type of tissue‐engineered corneal stroma material with a promising clinical application.

## 1 INTRODUCTION

Blindness has a serious impact on the human health and quality of life. According to the World Health Organization (WHO) report, about 61 million people are expected to go blind by 2050.^1^ Corneal damage is the fifth leading cause of blindness in the world, after cataract, refractive error, glaucoma, and age‐related macular degeneration.^2^ Cornea is the transparent structure of the outermost layer of the ocular that is very vulnerable to physical and chemical damage, and may lead to corneal opacity and even blindness. Currently, there are approximately 36 million blind people worldwide. Among them, corneal blindness can be restored by corneal transplantation. However, only 1 in 70 patients can ultimately undergo the graft transplantation.^3^

The cornea can be divided into five layers from anterior to posterior: epithelial layer, Bowman's membrane, stromal layer, Descemet membrane, and endothelial layer.^4^ The corneal stroma layer is approximately 500‐μm thick, accounting to a 90% of the entire corneal thickness, and consists of nearly 200 layers of regularly arranged collagen fibers. Unlike the epithelial layer, which has a strong regenerative capacity, the stromal layer is usually thought to lose its original cross‐linked structure due to changes in the diameter and gaps between the fibers of collagen formed by tissue repair after stromal injury, resulting in scarring and affecting vision.^5^ Currently, corneal transplants have evolved from full‐thickness penetrating keratoplasty (PKP) to partial lamellar keratoplasty (ALK). Compared to PKP, ALK has the advantages of fewer intraoperative complications, keeping the eye as intact as possible, and less possibility of postoperative graft rejection.^6^ Therefore, the development of corneal stromal equivalents needs an urgent study.

In recent decades, the amniotic membrane (AM), the acellular porcine corneal stroma (APCS), and hydrogel have made rapid progress in the research and development of corneal tissue engineering,^7^, ^8^, ^9^, ^10^ and a substantial amount of the research work has already reached the clinical practice^11^, ^12^, ^13^, ^14^, ^15^ However, the application of AM has many challenges, owing to its limited sources, potential infectious diseases, rejection after transplantation and ethical issues.^16^ The main difficulty faced in the use of the porcine‐derived xenograft as a donor in corneal transplantation is the immune reaction after transplantation, resulting in loss of transparency, neovascularization, and rejection. As a new functional material, hydrogels offer good biocompatibility, biodegradability, high water absorption, and water retention properties, which leads to its wide application in biomedical fields, including tissue engineering, drug delivery systems, wound dressings, and so on.^17^, ^18^ Although its physical, chemical, and mechanical properties are certain. It cannot simulate the composition and spatial configuration of the natural corneal stroma.^19^ Finally, a variety of natural and synthetic biomaterials have been used in corneal tissue engineering, still no material can perfectly satisfy all the characteristics of a tissue‐engineered cornea. Therefore, it is urgent to develop a novel tissue‐engineered corneal material.

Corneal graft materials need to possess several important properties, such as superior transparency, low immunogenicity, and excellent biocompatibility. The main component of the corneal stroma is collagen. Meanwhile, marine collagen has been found to possess an amino acid composition similar to the human type I collagen,^20^ as well as has advantages over mammalian collagen because of its excellent biocompatibility, low immunogenicity, low production cost, and lack of risk of transmission human susceptible viruses.^21^, ^22^ Currently, marine collagen has made significant advances in the fields of medical tissue engineering, drug delivery systems, and food medical and nutraceutical products.^23^, ^24^, ^25^ Squid mantles are rich in collagen (up to 29.3%) and mainly consist of type I collagen, and are abundant in essential amino acids.^26^ Interestingly, the squid mantle consists of epidermis and endothelium and a muscle layer sandwiched between the two, with a structural composition similar to that of the cornea.^27^ Squid is known to possess great application prospects in biomedicine and various tissue engineering fields. For example, squid chitin pen was used in the development of cartilage tissue engineering, which could promote the regeneration of rabbit cartilage.^28^ Squid outer skin was used to produce industrial‐grade films for medical usage.^29^ Decellularized squid mantle was used as urethral reconstruction materials.^30^ Based on the above findings, we speculated that squid mantle could be a potential corneal graft material. To the best of our knowledge, relevant research of squid in corneal tissue engineering has not been reported.

**SCHEME 1:**
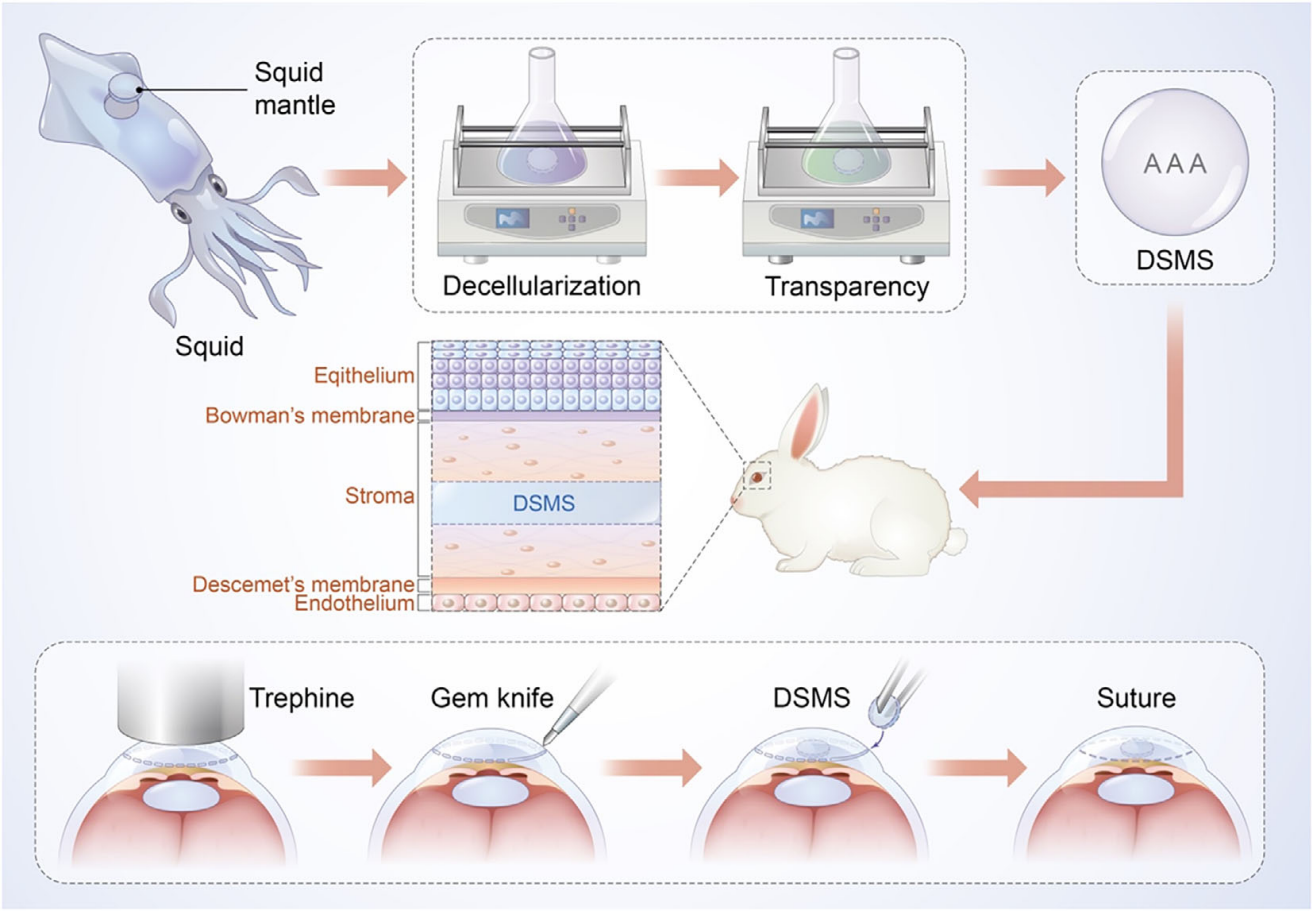
Overview of the development of DSMS for corneal regeneration. DSMS, decellularized squid mantle scaffold.

In the present study, we explored the feasibility of utilizing the squid mantle as a corneal stromal substitute in corneal transplantation (Scheme 1). First, the squid mantle was subjected to decellularization and transparency, and then the histological structure and chemical composition were further characterized. Subsequently, the in vivo and in vitro toxicity was evaluated. Finally, we implanted the decellularized squid mantle scaffold (DSMS) into the cornea of rabbit and observed its biocompatibility with the cornea. This study established a novel material for the construction of tissue‐engineered corneal stroma, which could possess potential applications in the field of cornea regeneration.

## 2 RESULTS

### 2.1 Transparency and characterization of DSMS

**FIGURE 1:**
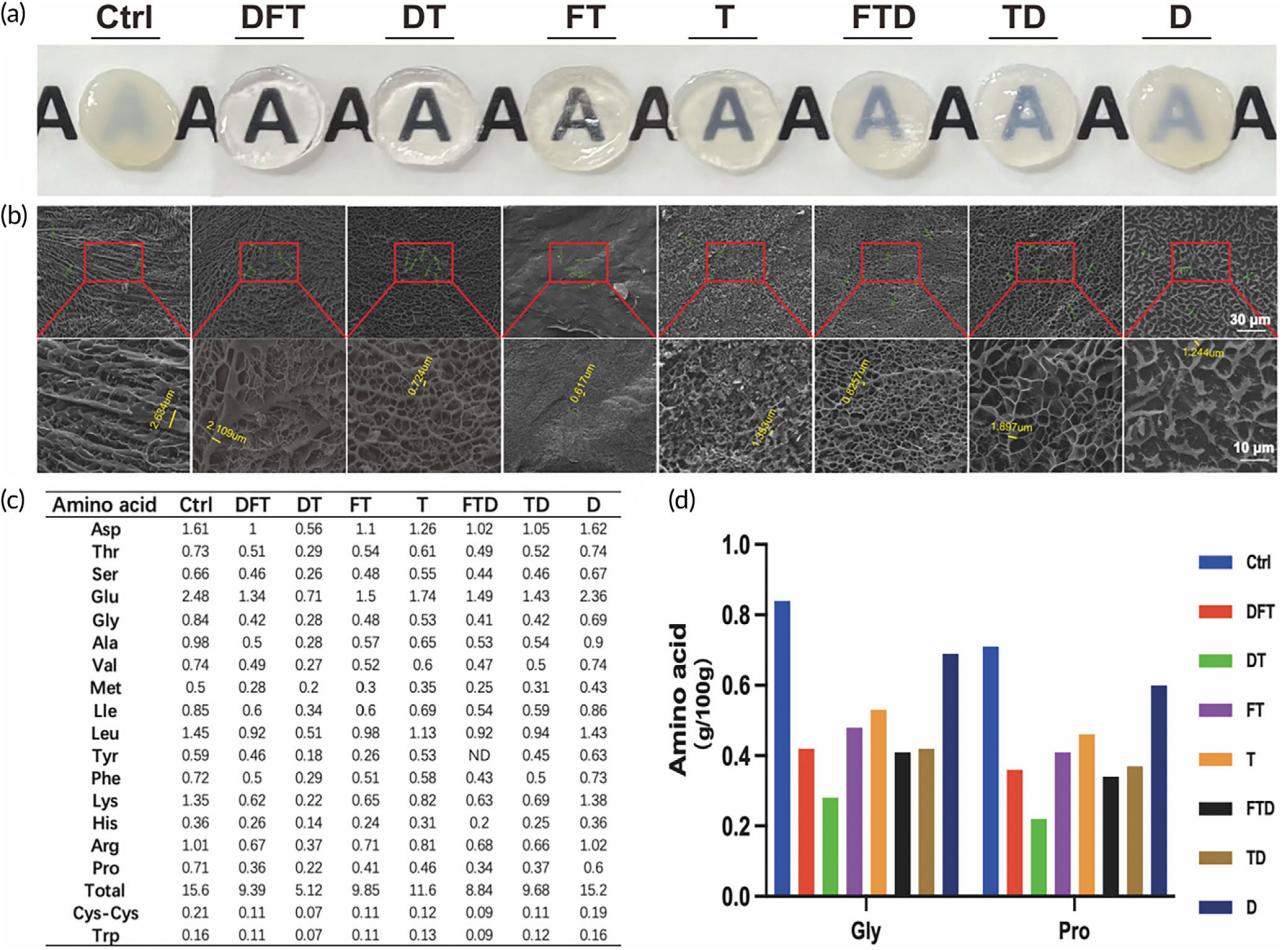
Transparency and characterization of DSMS. (a) Transparency of different groups of squid mantle. (b) Cryogenic transmission electron microscopy scanning photographs of squid mantle under different magnifications. (c) Amino acid contents of different groups of squid mantle. (d) Glycine and proline content in different groups. Ctrl, untreated group; D, decellularized group; DFT, decellularized and fixed and transparent group; DSMS, decellularized squid mantle scaffold; DT, decellularized and transparent group; FT, fixed and transparent group; T, transparent group, TD, transparent and decellularized group; TFD, transparent‐fixed‐decellularized group.

The material used for corneal transplantation should be transparent. Compared with the other six groups, the DFT and DT groups exhibited a better transparency (Figures 1a and S1). The translucency of the squid mantle was found to be improved after the decellularization and transparency processes.

Cryogenic transmission electron microscopy results (Figure 1b) show that the eight groups of materials exhibited a 3D grid‐like structure of acellular functional ECM. The untreated control group (Ctrl) presented a three‐dimensional structure with interwoven fibers and mesh, and the fibers were neatly arranged. Compared with the Ctrl group, the fiber structure of other groups was damaged to a certain extent. The fiber diameter of the DFT group was shorter than that of the Ctrl group, and the fiber pore size was larger than that of the Ctrl group, which was consistent with the view that the decellularization could damage the cytoskeleton. Besides, the fiber diameter of the DFT group was longer than that of the DT group, and the structure was more stable than that of the D group.

We examined the amino acid content in the eight groups of the squid mantle. The results (Figure 1c) showed that the squid mantle contained 18 amino acids. Glycine is the smallest amino acid, and the presence of glycine in every third amino acid residue is a key requirement for the superhelix structure of collagen.^31^ The stability of the helix is directly proportional to the amino acid content. The results (Figure 1d) indicated that the glycine and proline content decreased after the treatment, compared to the control group, but the content of the DFT group was higher than that of the DT group, indicating that the structure of the DFT group was more stable than that of the DT group.

### 2.2 Histological staining of DSMS

**FIGURE 2:**
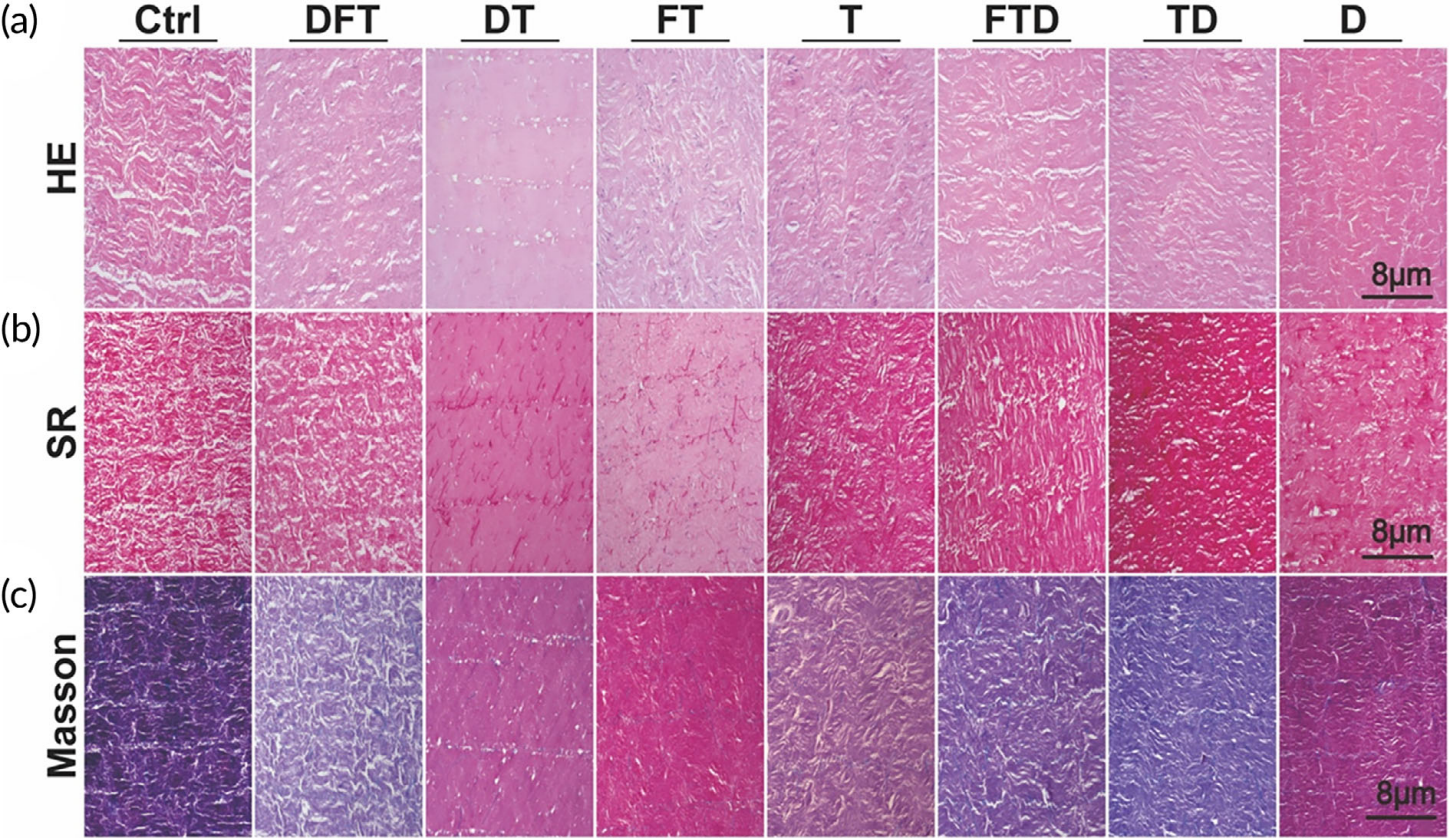
Histological staining of DSMS. (a) H&E staining of different groups. (b) Sirus Red staining of different groups. (c) Masson staining of different groups. DSMS, decellularized squid mantle scaffold; H&E, hematoxylin–eosin.

The surface texture of the squid mantle was smooth and dense. To further explore its internal structure, the paraffin sections of the tissue were stained with different histological staining methods. In the hematoxylin–eosin (H&E) staining (Figure 2a), myogenic fibers (red) in the Ctrl group were correctly arranged and nuclei (blue) in the fibers were clearly visible. Myogenic fibers in the DFT, FT, T, TD, and D groups were irregular compared with the Ctrl group, but many nuclei were visible in the DFT and DT groups. Myogenic fibers in the DT group were blurred and it was difficult to differentiate the fibrous tissue. The tissue gap in the FTD group was moderately extended, compared with that of the Ctrl group. In Sirius Red staining (Figure 2b), myogenic fibers (red) were clearly seen in the Ctrl, DFT, T, FTD, TD, and D groups, but the myogenic fibers in DT and FT groups were blurred and indistinguishable, and some small fragments appeared. Meanwhile, in Masson staining (Figure 2c), the squid mantle tissues of the Ctrl group were closely connected with the myocollagen (blue) and myogenic fibers (red), and it was seen that the myocollagen was evenly distributed in the tissues (showing a purple color). The color of the other groups was between blue and red, and we speculated that it could be due to a change in the ratio between the myocollagen and myogenic fibers due to the decrease in the binding between them or a decrease in the myocollagen in the tissues after different treatments of the squid mantle.

### 2.3 Effects of decellularization of squid mantle using different methods

**FIGURE 3:**
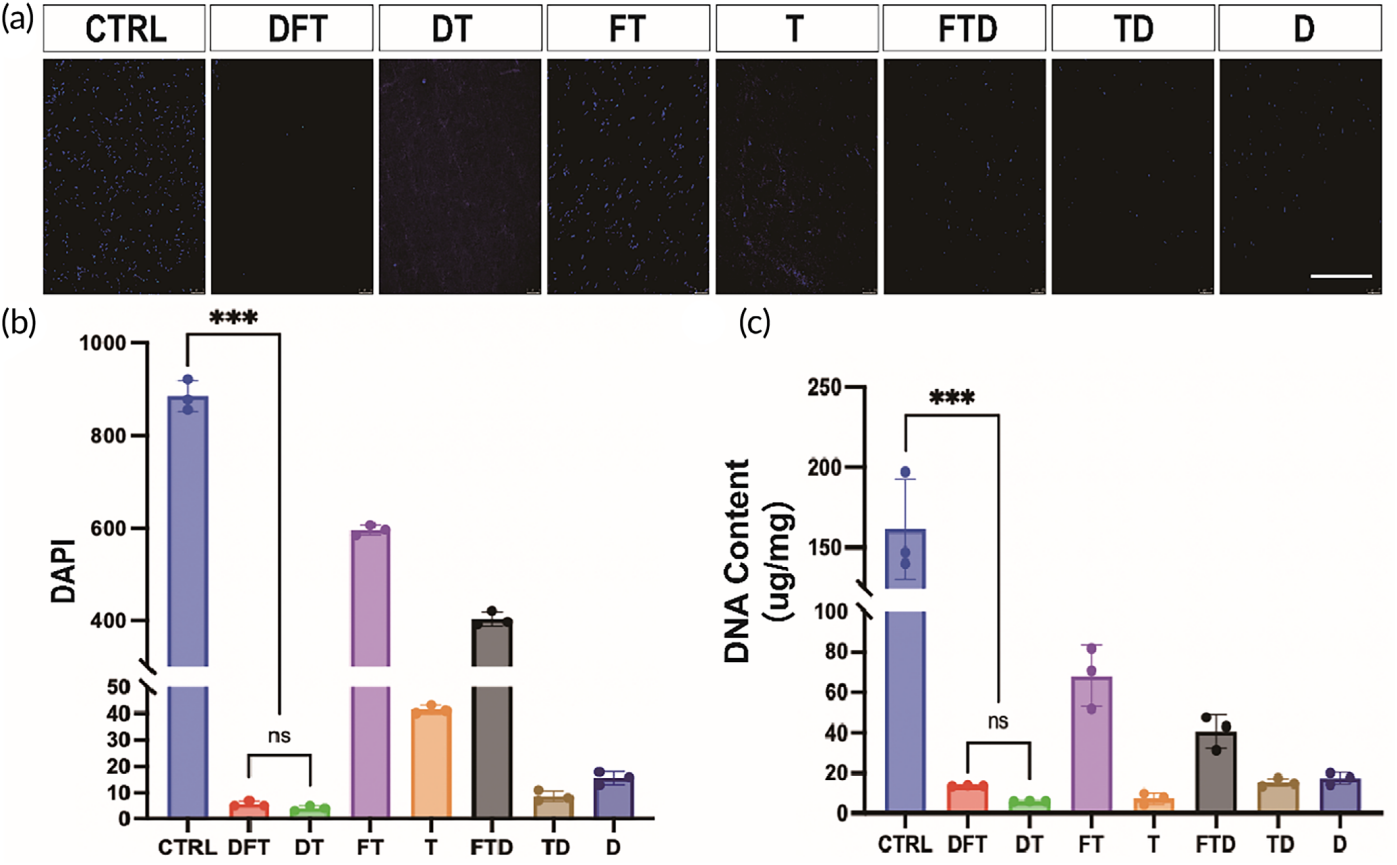
Effects of different methods on decellularization of squid mantle. (a) DAPI staining images of different groups. (b) Quantitative analysis of DAPI staining in different groups. (c) Quantitative analysis of the DNA content in different groups (*p < 0.05, **p < 0.01, ***p < 0.001).

To verify the efficiency of different method of decellularization, the squid mantle was subjected to staining with DAPI for evaluating the nuclear number (Figure 3a). The results showed that the acellular effect in DFT and DT groups was significantly higher than that of the Ctrl group (Figure 3b). Furthermore, the experimental results of the quantitative analysis of DNA content were consistent with that of the DAPI staining (Figure 3c). In the present study, triton X‐100 was one of the main components of the CUBIC clearing method. As a result, the double washing by SDS and triton X‐100 could better remove the nuclei. This shows that the proposed method was very efficient in the decellularization process. Figure S2 shows that the Ctrl and D groups exhibited deformation at 12 h, and the phosphate‐buffered saline (PBS) solution became significantly turbid. On the third day, the tissue was swollen, which then gradually degraded and became smaller in size. In the DT, T, and TD groups (without fixation), the tissue size began to slowly decrease at 12 h, but basically remained round. The DFT, FT, and FTD groups maintained relatively stable and regular shapes from beginning to end, indicating that fixation made the tissues more compact and firmer. In particular, PBS in the DFT group remained clear all the time, indicating that the DFT group had the strongest resistance to collagenase.

### 2.4 In vitro and in vivo biocompatibility assessment of DSMS

**FIGURE 4:**
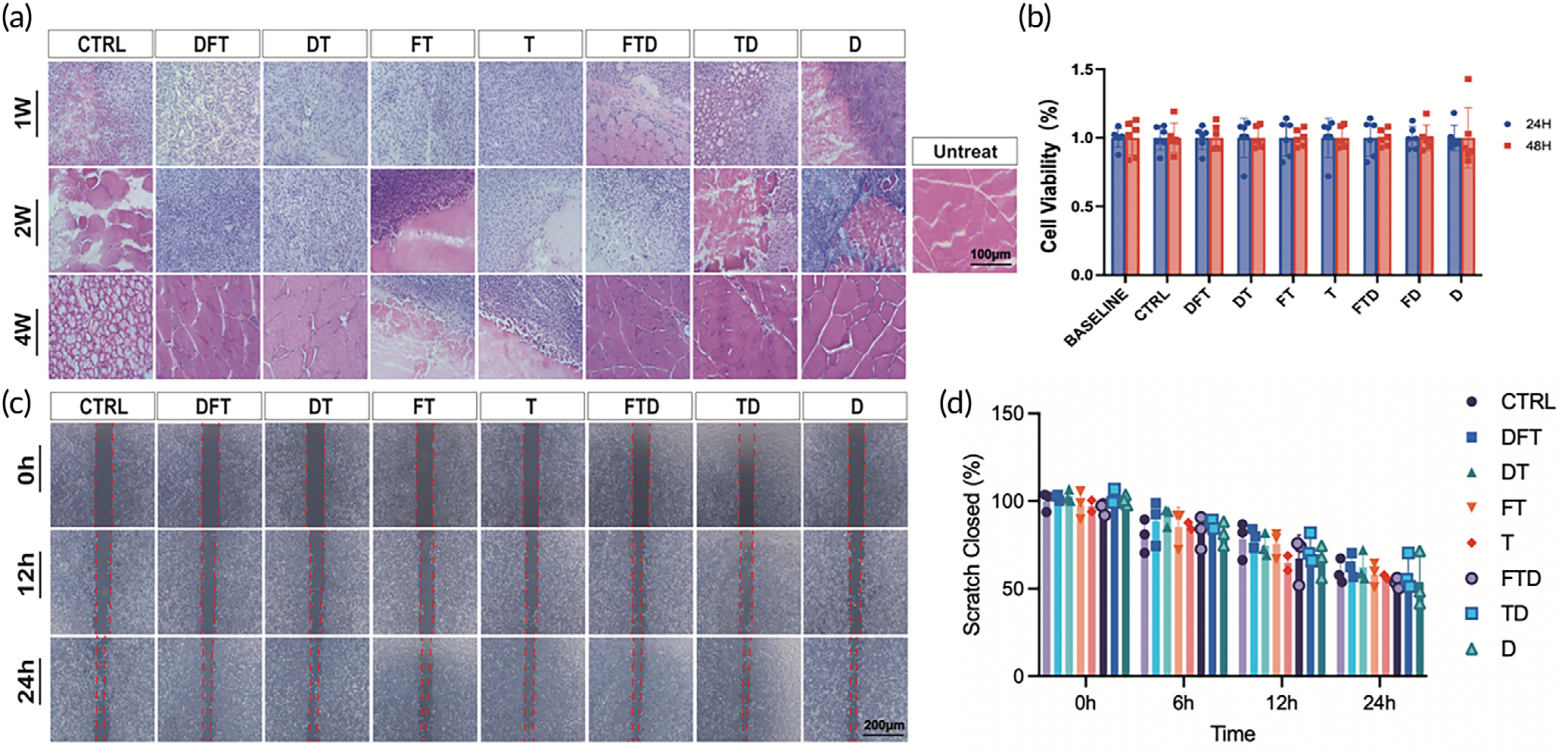
In vitro and in vivo biocompatibility of DSMS. (a) H&E staining of rat muscle tissue (implantation area) at 1, 2, and 4 W after dorsal muscle implantation (Untreat represents normal muscle tissue and Ctrl represents the squid mantle without treatment). (b) Viability of corneal epithelial cells in different leaching liquor group on 24 and 48 h by CCK‐8 assay. (c) HCE cells were subjected to wound healing assay in 0, 12, and 24 h. Red lines highlight the migration front. (d) Quantification of wound healing assay. CCK‐8, Cell Counting Kit‐8; DSMS, decellularized squid mantle scaffold; H&E, hematoxylin–eosin; HCE, human corneal epithelium.

In vitro Cell Counting Kit‐8 (CCK‐8) tests of the leaching liquor from DSMS were performed to observe the effects of DSMS on the proliferation and growth of corneal cells. It was observed that there were no significant differences in the corneal epithelial cell viability between all groups compared with the baseline (Figure 4b). Furthermore, the live‐dead staining assay was also performed to discriminate between the live and dead cells. In accordance with the CCK‐8 tests, there was no significant increase in the number of dead cells in each group when compared to the Ctrl group (Figure S4). The results indicated that DSMS was biocompatible with the corneal cells.

Rat dorsal muscle transplantation was performed to determine the immunogenicity of DSMS. To avoid the displacement of DSMS due to the loose rat skin, DSMS was transplanted to the left dorsal muscle of the rat.^32^ No delayed wound closure, postoperative infection, or suture‐related complications were noted during the visual inspection. Histological staining results (Figure 4a) showed that the inflammatory cells infiltrated around the samples in all the groups at 1 and 2 weeks postoperatively. A small ball was seen at the site of transplantation in several groups, and inflammatory cells were seen at the site of transplantation on the tissue sections. We speculate that the reason for this phenomenon may be due to the inadequate elution of the decellularized reagents. Detergents used for decellularizing cells are known to cause immunologic reactions, with studies showing that residual SDS in biocompatible materials could elicit host immune response after implantation due to the difficulty in its complete removal.^33^ We measured the residual SDS content of the materials, and found the SDS content was lower in the DFT group when compared to the D group (Figure S3). An exacerbation of inflammation due to the mechanical irritation of the double suture certainly cannot be ruled out. Besides, the size of DSMS transplanted into muscle in this study was much larger than the size of DSMS transplanted into the cornea. As a result, we presume that these factors led to a strong inflammatory reaction at the graft site; however, after 2 weeks, the inflammation gradually subsided. In the fourth week sample, the inflammatory cells disappeared, especially in the DFT and DT groups, and the peripheral muscle structure also recovered like the normal muscle. In particular, the implants were degraded in the control group, leading to a loss of the normal muscle structure, and became vacuolated. All rats survived during the postoperative follow‐up. Our results indicate that although DSMS are heterogeneous biomaterials, they have excellent histocompatibility.

The wound‐healing assay showed that the distance between the cell scratches from each group was equal to that of the control group at every time point (Figure 4c,d). It indicated that DSMS was non‐toxic to the cells and could promote their normal proliferation and migration. Besides, immunofluorescence staining with ZO‐1 was further conducted, and the results showed that DSMS did not cause any phenotypic changes (Figure S5).

### 2.5 Postoperative observation by slit lamp

**FIGURE 5:**
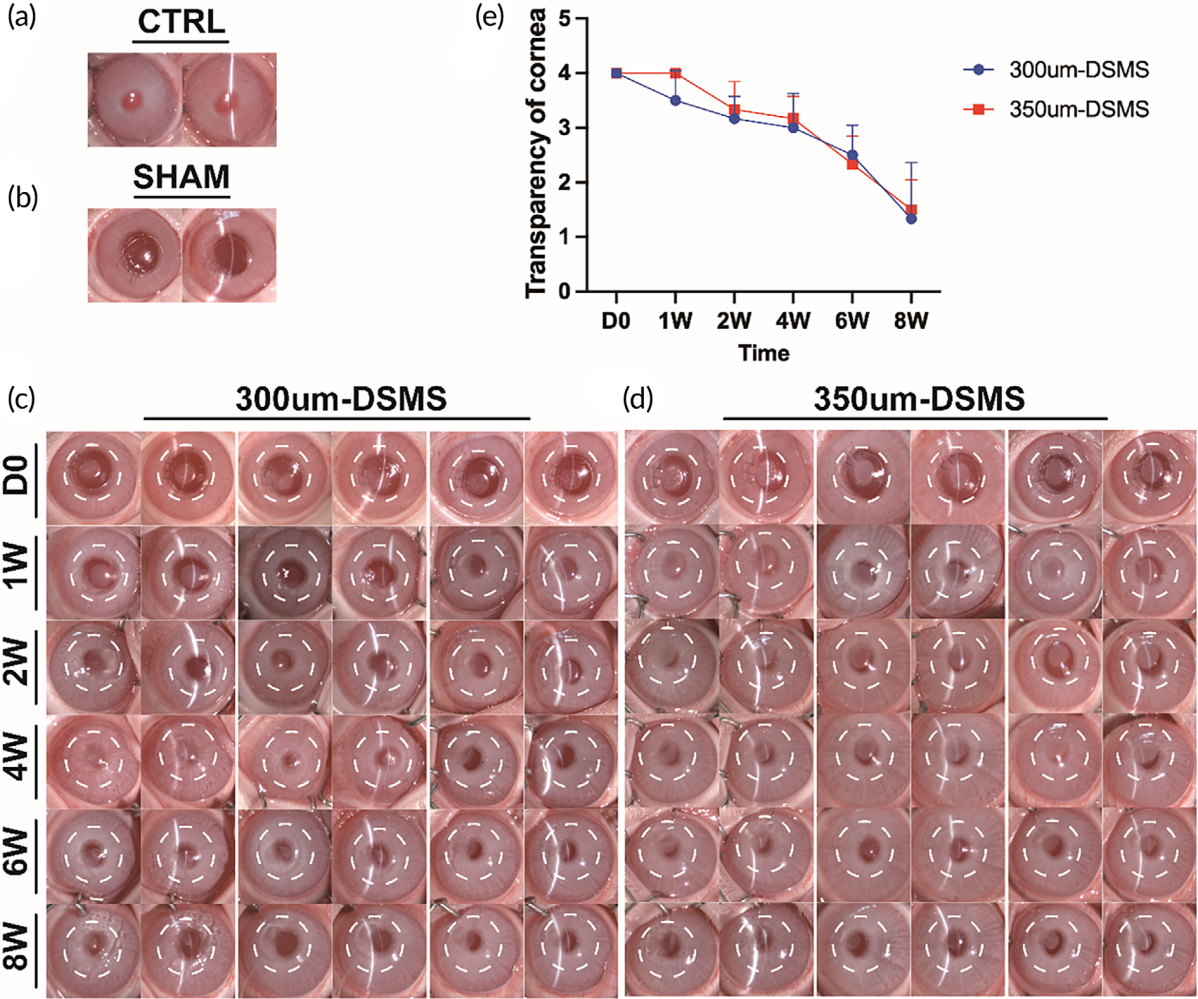
Slit lamp photographs of intra‐stromal implantation of DFT‐DSMS in rabbits. (a) Slit lamp images of the cornea of normal rabbit. (b) Slit lamp images of the cornea in sham operation. (c) Slit lamp images of the cornea (with a slit at the central of cornea) at 0, 1, 2, 4, 6 and 8 weeks after the implantation of DFT‐DSMS (with the thickness of 300 μm). (d) Slit lamp images of the cornea (with a slit at the central of cornea) at 0, 1, 2, 4, 6 and 8 weeks after the implantation of DFT‐DSMS (with the thickness of 350 μm; white circle represents the implantation position of DFT‐DSMS). (e) Quantitative statistics by transparency of cornea for different groups during observation (right eye was selected in the study, *n* = 6/group). DFT, decellularized and fixed and transparent group; DSMS, decellularized squid mantle scaffold.

Rabbit corneal interlamellar implantation was performed to evaluate the condition of cornea after DSMS implantation. Based on the above results, the DSMS using DFT method was found to be better for the corneal stromal implantation graft in New Zealand rabbits. Figure 5b shows that in the sham operation group, the cornea remained transparent throughout the whole follow‐up period, and corneal edema recovered rapidly, when compared to the normal cornea (Figure 5a). In the DSMS implantation group, the cornea seemed obscure at the beginning, which could be due to the absorption of water from the material during the preoperative preservation (Figure 5c,d). However, during the follow‐up observations, the transparency of cornea gradually increased as the DSMS fitted to the cornea and no epithelial defect or corneal melting was noted. To avoid the irritation, corneal sutures were removed after 1 week of the operation. All the animals survived without any complications and infections like immune rejection, conjunctival edema, congestion, and neovascularization. To further explore the transparency of cornea with different thicknesses of DSMS after transplantation, we performed the evaluation of corneal transparency criteria (Table 1). It was found that the transparency of cornea in both DSMS groups began to recover at 4 weeks after transplantation, and there were no adverse reactions noted during the observation period (Figure 5e).

**TABLE 1:** Transparency criteria for cornea during post‐operation observation.

	Grade 0	Grade 1	Grade 2	Grade 3	Grade 4
Area of corneal opacity	0	1%–25%	26%–50%	51%–75%	76%–100%
Density of corneal opacity	Not cloudy	Slight cloudiness, pupil visible	Cloudy, mostly pupil discernible	Moderate cloudiness, partial pupil discernible	Cloudy, opacity, pupil invisible

### 2.6 Postoperative observation by anterior segment optical coherence tomography and swept‐source optical coherence tomography angiography

**FIGURE 6:**
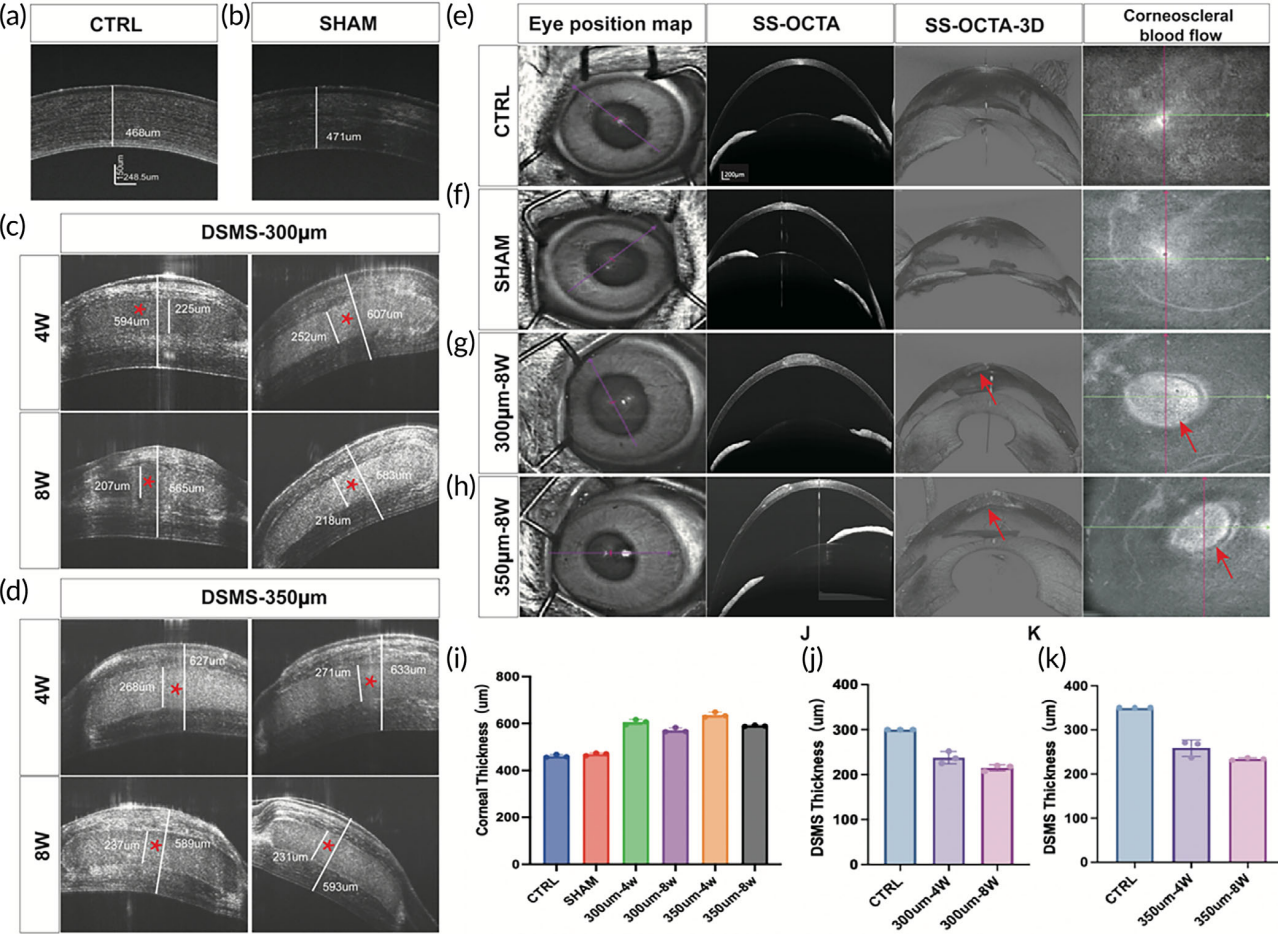
Long‐term follow up of AS‐OCT and SS‐OCTA images of the DSMS implantation of corneas in rabbits. (a) AS‐OCT images of the cornea of normal rabbits. (b) AS‐OCT images of the cornea of rabbits undergoing sham surgery. (c) AS‐OCT images at fourth and eighth weeks after implantation of DSMS (with the thickness of 300 μm). (d) AS‐OCT cross section images at fourth and eighth weeks after implantation of DSMS (with the thickness of 350 μm) (* represents the implantation position of the DSMS). (e) SS‐OCTA images of the cornea of normal rabbits. (f) SS‐OCTA images of the cornea of rabbits undergoing sham surgery. (g) SS‐OCTA images at fourth and eighth weeks after implantation of DSMS (with the thickness of 300 μm). (h) SS‐OCTA images at fourth and eighth weeks after implantation of DSMS (with the thickness of 350 μm; red arrow represents the implantation position of the DSMS). (i) Quantitative statistics of corneal thickness (*n* = 3/group). (j) Quantitative statistics of DSMS thickness (with the thickness of 300 μm, *n* = 3/group). (k) Quantitative statistics of DSMS thickness (with the thickness of 350 μm, *n* = 3/group). AS‐OCT, anterior segment optical coherence tomography; DSMS, decellularized squid mantle scaffold; SS‐OCTA, swept‐source optical coherence tomography angiography.

Anterior segment optical coherence tomography (AS‐OCT) and swept‐source optical coherence tomography angiography (SS‐OCTA) were used to measure the corneal thickness, graft thickness, and corneoscleral blood flow after transplantation. It was observed that in the sham surgery group, the corneal healing was faster, the corneal stromal layer density was uniform, and the boundary between the stromal layer and epithelial layer was obvious as the normal cornea (Figure 6a,b). AS‐OCT images showed that all DSMSs were intact in the corneal stroma. The results indicate that there was no corneal extrusion in the DSMS transplantation group, no complications were noted during the follow‐up period, and DSMS and stroma were well fused (Figure 6c,d). After the effects of enzymatic digestion, intraocular pressure, and implant dehydration, the thickness of the entire cornea and DSMS stabilized at 8 weeks without material detachment (Figure 6i–k). The 300‐μm DSMS was clear at 4 weeks and maintained this transparency until 8 weeks. About 350‐μm DSMS showed some light reflection (corneal clouding) at 4 weeks and was still present at 8 weeks. At Week 8, we monitored the corneoscleral blood flow using SS‐OCTA, and the experimental results (Figure 6e–h) showed that no traces of blood flow were observed in all rabbit corneas, and the experimental results further confirmed the absence of neovascularization in the transplanted DSMS.

### 2.7 Postoperative observation by in vivo confocal microscopy

**FIGURE 7:**
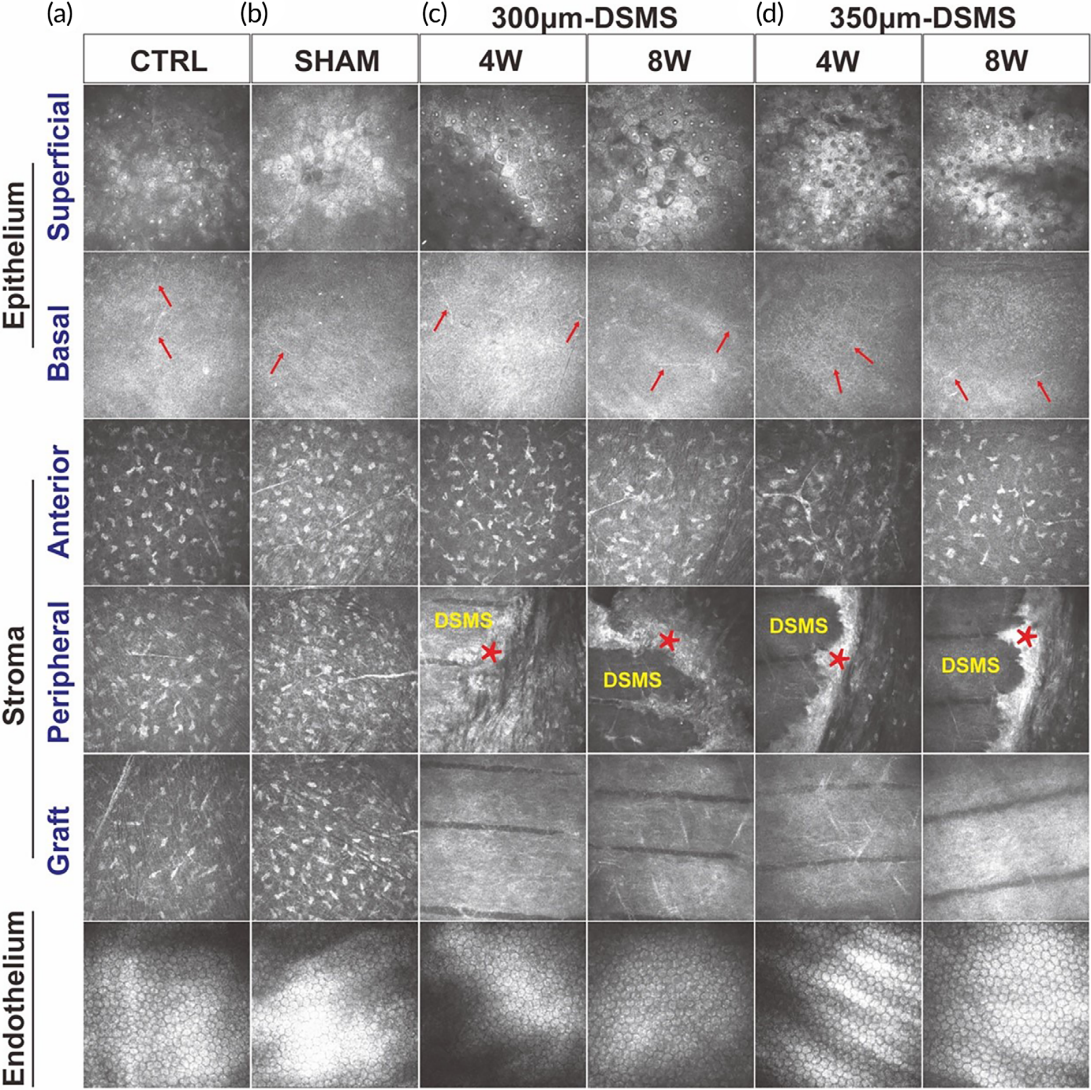
In vivo confocal microscopy images from different layers of cornea. (a) Corneal structure of normal rabbits at the cellular level (with an image of same depth as graft from implantation of DSMS). (b) Corneal cellular structure and morphology of rabbits receiving sham surgery (with an image of same depth as graft from implantation of DSMS). (c) Corneal cellular structure and morphology from each layer of cornea at fourth and eighth weeks after implantation of DSMS (with the thickness of 300 μm). (d) Corneal cellular structure and morphology from each layer of cornea at fourth and eighth weeks after implantation of DSMS (with the thickness of 350 μm) (red arrows indicate the sub‐basal nerve and red star represents the DSMS‐Stroma boundary). All images are 400 × 400 μm^2^. DSMS, decellularized squid mantle scaffold.

In vivo confocal microscopy was performed to assess the morphology of the implants and all cellular layers of the surrounding host corneal tissue.^34^ The results from Figure 7a show a normal corneal structure in the control group and no obvious corneal abnormalities in the sham surgery group (Figure 7b). Similarly, the epithelial cell layer exhibited a more regular morphology with a complete structure and a round bright nucleus visible in the center of the cell (Figure 7c,d). More importantly, regeneration of the subbasal nerve can be observed. Corneal nerve is essential for wound healing and long‐term corneal homeostasis. Four weeks after the operation, the sub‐basal nerve was seen under the central epithelium of the cornea in each group, indicating that the sub‐basal nerve plexus was preserved. This proved that DSMS exhibited a good histocompatibility with the rabbit cornea, and did not cause rejection. In parallel, the stromal layer exhibited the orderly collagen fibers of same diameter, short rod‐shaped corneal stromal cells, and occasional branches of nerve fibers. All the endothelial cell layers exhibited a regular flattened hexagonal morphology, and there were tight connections between cells, with a three‐dimensional appearance. It was also evident that the DSMS was flat and smooth in the graft layer. No inflammatory cells (with high‐bright shadow) were observed throughout the period. The results revealed that the DSMS exhibited a good biosafety profile with the corneal cells.

### 2.8 Histological analysis of cornea after implantation in rabbits

**FIGURE 8:**
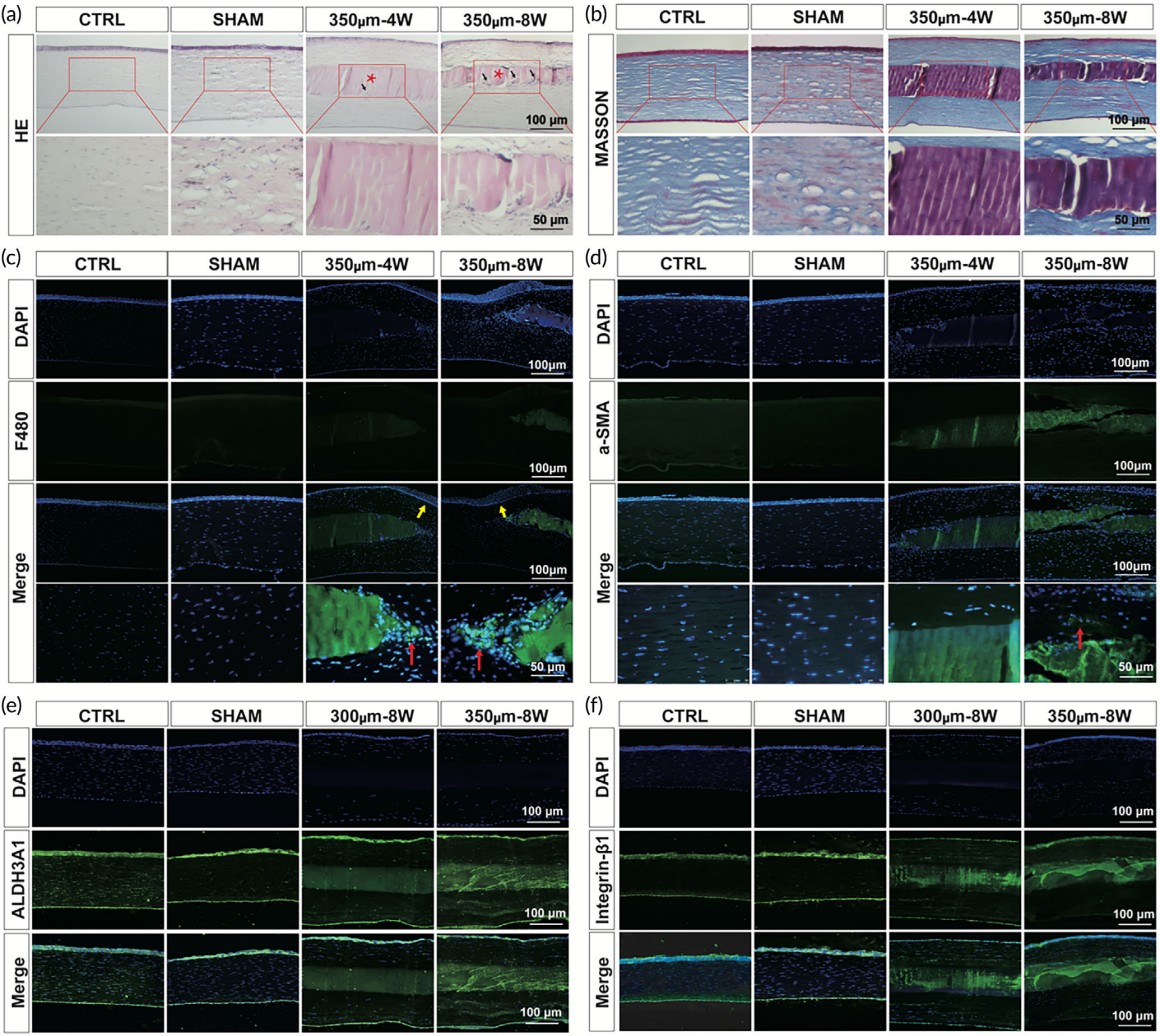
Staining of paraffin sections after the fourth and eighth weeks of surgery in rabbits. (a) H&E staining (black arrow indicates corneal stromal cells and red stars represent DSMS). (b) Masson staining. (c) Immunofluorescent staining for F480 (green) and nuclei (blue) in keratocytes (red arrow indicates the macrophage cells and yellow arrows indicate the thickened epithelial layer). (d) Immunofluorescent staining for α‐SMA (green) and nuclei (blue) in corneal stroma (red arrow indicated the α‐SMA positive). (e) Immunofluorescent staining for ALDH3A1 (green) and nuclei (blue) in keratocytes. (f) Immunofluorescent staining for Integrin β1 (green) and nuclei (blue) in keratocytes. DSMS, decellularized squid mantle scaffold; H&E, hematoxylin–eosin.

Histological analysis of cornea was performed to verify the effects of DSMS on the corneal stroma reconstruction. In the H&E staining results (Figure 8a), the structure of all layers of the cornea was clearly observed in the Ctrl group, especially the epithelium, stroma, and endothelium. In the sham operation group, the corneal structures were clearly visible, and the surgical incision had healed without forming a scar. In the DSMS implantation group, the corneal structure stayed normal, with well‐defined tissue structure in all layers, seamless adhesion of the graft to the host cornea, and neatly arranged stromal fibers, indicating no immune rejection. The implanted DSMS was clearly identified by H&E staining. Tissue sectioning sometimes results in DSMS separation from the corneal stroma; however, the areas of adhesion tissue were still evident, and all implant materials remained intact in the cornea of rabbit eyes. Sporadic signs of corneal stromal cells (black arrows) starting to invade DSMS were observed in the fourth week after transplantation, with many cells invading by the eighth week. We suspect that this may be an indication that the degradation of DSMS promotes the migration of corneal stromal cells to the vacant site.

Masson study was performed to stain the collagen fibers. Masson staining results showed that the corneal stromal fibers in the sham operation group were arranged neatly, and no scar was observed. In the implantation group, it was observed that the rabbit corneal stromal fibers were arranged neatly, and the graft was closely fitted to the stroma, indicating that DSMS exhibited a good histocompatibility with cornea (Figure 8b). and no corneal protrusion was caused due to the transplantation.

The F480 immunofluorescence staining was performed to observe whether the DSMS caused inflammatory reaction in the host cornea. The staining results (Figure 8c) show that there were no differences between the sham operation group and the normal group, and almost no inflammatory cells were visible. In the DSMS group, the corneal stroma was thicker and there was thickened epithelium at the edge of the implantation area. The inflammatory cell marker F480 was absent in the rabbit corneal stroma and DSMS, while only the margins at the junction of DSMS and corneal stroma showed varying degrees of F480 positive. This is consistent with the macrophage‐associated peripheral implant edge remodeling observed by other investigators.^13^, ^14^

We performed immunofluorescence staining for the myofibroblast marker α‐SMA (Figure 8d), keratocyte marker ALHD3a1 (Figure 8e), and integrin β1 (Figure 8f) to evaluate the corneal damage repair after DSMS transplantation. The results showed that fibrosis occurred mainly in the anterior corneal stroma area connecting the edge of the DSMS and host. No significant fibrosis was found in the stroma, and fibrotic repair was limited to the peripheral rim. This is consistent with the AS‐OCT results. AS‐OCT results showed strong light reflection around the graft at 350 μm—8 W. In the post‐transplant corneal stroma, equal levels of Aldh3a1 and integrin β1 were noted, when compared to normal corneas.

## 3 DISCUSSION

Although varieties of natural and synthetic polymer scaffolds are widely used for tissue‐engineered cornea, the original scaffolds of decellularized animals are more valuable due to their excellent biocompatibility, biodegradability, and low immunogenicity.^35^, ^36^ APCS is the commonly used decellularized corneal stroma material, nevertheless, recent studies have revealed early complications such as sterile keratolysis, corneal calcification, and persistent epithelial defects, when APCS is used for corneal transplantation.^15^, ^37^, ^38^ Recently, Rafat et al. developed a cell‐free engineered corneal tissue, bioengineered porcine construct, double cross‐linked (BPCDX) corneal tissue material derived from purified medical‐grade type I porcine collagen as corneal stromal implants.^13^ Furthermore, the original porcine corneal stroma material also has several problems, for instance, long reproduction period, high production cost, the risk of zoonotic diseases, and religious restrictions. Meanwhile, synthetic polymer scaffolds like hydrogel show a massive potential for tissue‐engineered cornea in animal studies; however, a further validation for safety is needed before clinical application. Therefore, it is necessary to develop a novel tissue‐engineered corneal stromal substitute, to enrich the selectivity of materials.

Our findings provide evidence that the intracorneal stromal implantation of a decellularized marine‐derived colloidal material is a safe and feasible process, wherein the DSMS transplants not only remained relatively clear, but also promoted the corneal stromal regeneration along with enhancing the thickness of the corneal stroma. In recent years, the combination of tissue engineering and marine biomaterials has provided rich resources for the preparation of artificial organs or tissue substitutes.^39^ Ueda et al. reported that decellularized and decalcified fish scale‐derived collagen matrix met the basic features to act as artificial corneas to replace the donor corneas.^40^ This showed a great potential of non‐corneal‐derived marine materials in the field of corneal tissue engineering.

The cornea is the only transparent organ in the body and being the main component structure of the refractive system, results in a high demand for the transparency of the scaffolds. Multiple materials based on decellularization methods did not achieve the ideal transparency for artificial corneas according to the literature.^41^, ^42^ Therefore, improving the transparency, while ensuring a low immunogenicity of the scaffold is indeed a challenging task.^15^ Tissue clearing technology was originally created to make the biological samples transparent, to observe the 3D visualization structure of tissues, and to provide a new platform for further exploration of human diseases.^43^ DSMS was prepared by using a combination of the SDS decellularization method and the modified CUBIC method.^44^ The CUBIC method used water‐soluble reagents for clearing the tissue. Although the transparent effect was not as potent as the hydrophobic method, the former has a higher level of biocompatibility, biosafety, and protein preservation. The hydrophilic reagent allows the protein to form hydrogen bonds with surrounding water molecules, which helps to preserve the three‐dimensional structure of the tissue components.^45^ Our data showed that the CUBIC technique resulted in transparent squid mantles, which further demonstrated the feasibility of using a graft material of non‐corneal source. To our knowledge, this is the first report of combining decellularization and clearing methods and using them to make tissue‐engineered corneal stromal equivalents. However, it has to be admitted that even the mildest method may cause some damage to the tissue structure. According to our findings, the damage to the squid mantles can be reduced after the modification and fixation process.

Collagen materials have been widely used as scaffold materials in the field of tissue engineering and collagen‐based materials have been implanted in humans in clinical trials, owing to its excellent biocompatibility, biodegradability, low immunogenicity, and cell adhesion.^14^, ^32^, ^46^, ^47^, ^48^, ^49^ While most common collagen is derived from terrestrial animals, over the past few years, there has been an increasing interest in other sources of collagen due to the risk of zoonotic disease transmission.^21^ Studies have shown that marine collagen has certain advantages over the terrestrial collagen, such as abundance in source and no risk of disease transmission. It has also been demonstrated that squid‐derived collagen showed a less antigenic response than the terrestrial collagen to support cell growth.^50^ However, a potential limitation is the immunogenicity of the decellularized material may arise due to the incomplete removal of the cellular components and DNA during the decellularization process. Minute quantity residues of cellular components or chemicals can trigger an immune response. So far, most of the tissue engineering products from animals (pigs or squid) have not been genetically modified to neutralize genes that could trigger an immune reaction in humans and some immunogenicity is expected, especially if the corneal immune amnesty has been compromised by the initial disease (since rabbits employed in this study were healthy, but in case of humans, they usually suffer from corneal disease).

The 350‐μm DSMS may not be optimal in terms of in vivo optical transparency and maintenance of total corneal thickness. However, it demonstrates the ability to substantially thicken the corneal stroma using thicker DSMS for the treatment of conditions such as corneal dilatation, ulceration, and cone corneas. In addition, considering that the implant is subject to enzymatic degradation, intraocular pressure, and implant dehydration in the cornea, it was found that DSMS degraded at a certain rate in the first 8 weeks postoperatively, after which the corneal thickness tends to stabilize. This precisely shows that DSMS can be adjusted in size and thickness according to the surgical needs and can be better applied to different lesion scopes and patients with a humanized and personalized approach. Squid is an abundant marine resource, and by the method used in this study, hundreds of corneal implants can be prepared from a normal‐sized squid, minimizing production costs, and alleviating the global shortage of donor corneas. However, it must be acknowledged that our study has some limitations. First, this trial used DSMS in only two thicknesses for the preliminary study. Second, our study was limited to interlamellar corneal grafting only, where the implant was free from mechanical pulling of sutures. The corneal epithelial‐stromal and implant interaction were not involved here as the intracorneal stromal implantation of DSMS only reflected corneal stromal inflammation of grafts. However, future plan was conducted for the corneal lamellar grafts to observe epithelial‐stromal and implant interaction. We are constantly exploring ways to further improve the overall performance of the DSMS for better and more comprehensive clinical applications.

## 4 MATERIALS AND METHODS

### 4.1 Preparation of DSMS

**SCHEME 2:**
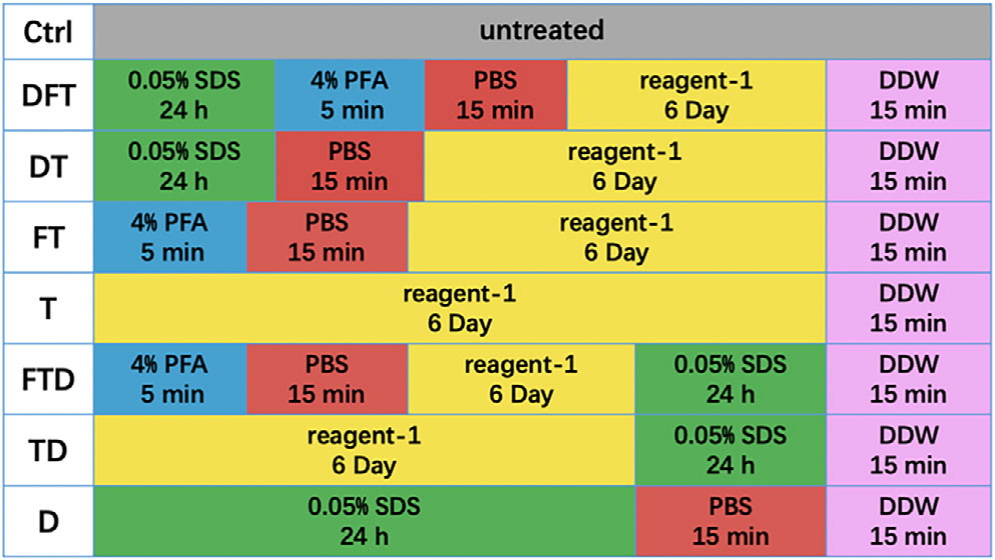
Preparation of the DSMS. DSMS, decellularized squid mantle scaffold.

The whole raw squid was obtained and the head, chitin pen, transparent strip, and viscera inside the squid were removed. The outer membrane was peeled off. Deuterium‐depleted water (DDW) was used to clean the mantle several times at 4°C. Next, the mantle was cut to 10 × 10 mm for each piece, followed by cutting into 3 mm thickness and washing with DDW three times. The squid mantle tissues were divided into eight groups: untreated group (Ctrl), decellularized and fixed and transparent group (DFT), decellularized and transparent group (DT), fixed and transparent group (FT), transparent group (T), transparent‐fixed‐decellularized group (TFD), transparent and decellularized group (TD), and decellularized group (D). Eight groups of squid mantles were performed using an immersion method in a shaker at 37°C with a shaking speed of 100 rpm, as shown in Scheme 2.

Reagent required for 0.5% SDS method: 5 g of sodium dodecyl sulfate (SDS) powder was dissolved in 1000 mL DDW.

Reagents required for CUBIC method: 80% Quadrol: 500 g Quadrol (Sigma‐Aldrich) was subjected to 125 g deionized water. ScaleCUBIC‐1 (also known as reagent‐1): 125 g urea (Solarbio) and 156 g 80% Quadrol were added in 144 g deionized water with heating and stirring, dissolved fully and then cooled to room temperature (RT), then added 75 g of Triton X‐100 (Solarbio), dissolved fully and then cooled to RT.^43^

### 4.2 Transparency test

Each set of samples was cut with a 5‐mm trephine and placed on an A‐card to observe the transparency and imaged by a digital camera.

### 4.3 Cryo‐transfer transmission electron microscopy

The samples were swiftly frozen and fractured by liquid nitrogen under high vacuum conditions. Subsequently, the samples were subjected to sublimation and conductivity spraying under vacuum at −90°C. The samples were then transferred to a scanning electron microscope cooling stage (down to −160°C) by a freeze transfer system in the next step. The microstructure of each group of samples was observed by cryogenic transmission scanning at an accelerating voltage of 3.0 kV.

### 4.4 Amino acid composition and total protein content

The amino acid content was testified in accordance with the guidelines of the national standard (National Food Safety Standards‐Determination of Amino Acids in Food; GB 5009.124‐2016). Briefly, the squid mantle was hydrolyzed with hydrochloric acid to liberate the amino acids. Subsequently, the content of 18 amino acids, including aspartic acid (Asp), threonine (Thr), serine (Ser), glutamic acid (Glu), proline (Pro), and glycine (Gly), was determined by post‐column ninhydrin derivatization cation exchange chromatography. Finally, the amino acid content was determined by visible light spectrophotometer at wavelength 570 and 440 nm. The test was conducted by SGS‐CSTC Standards Technical Services Co., Ltd.

### 4.5 Paraffin‐embedded section

The samples were cut into squares of size 10 × 10 × 3 mm, soaked, and dehydrated sequentially in graded ethanol (70%, 80%, 95%, and 100%) for 1 h each time. Then, xylene was used for transparency two times each 1 h. Then the material was infiltrated with paraffin. The solidified paraffin blocks were installed on the clamping table of the slicer to cut into slices with a thickness of 10 μm.

### 4.6 Histological staining for DSMS

#### 4.6.1 H&E staining

Initially, the sections were put into oven to dissolve the paraffin and the paraffin was removed with xylene and alcohol. Sections were stained with hematoxylin for 5 min, washed with water and differentiated by 1% hydrochloric acid alcohol solution, and washed in running water until they turned blue. Next, sections were dyed using eosin for 40 s and rinsed with water, dehydrated with gradient alcohol concentrations and rinsed in xylene. Finally, the sections were sealed with neutral gum and observed under a light microscope (Eclipse E400 with DS‐Fi1, Nikon).

#### 4.6.2 Sirus red staining

The sections' preparation steps were same as described in the H&E staining. Sections were stained with Sirius red droplets for 1 h and washed under running water to remove excess dye. Then, the sections were stained with hematoxylin for 8 min and washed under running water. The sections were dehydrated in ethanol and immersed in xylene. Finally, they were sealed using neutral gum and observed under a light microscope (Nikon).

#### 4.6.3 Masson staining

The Masson Trichrome Staining Kit (product code: R21866) was used. Briefly, the paraffin from the paraffin sections was removed using gradient alcohol and then dehydrated in xylene. The sections were stained with Wiegert's iron hematoxylin, differentiated in acid ethanol solution, then with Masson blue solution, and then washed with DDW. Subsequently, the sections were stained with Lechon red magenta, and washed with a weak acid solution and phosphomolybdic acid solution before staining in aniline blue solution. This was followed by gradient alcohol dehydration, xylene transparency, sealing using neutral gum, and observation under a light microscope (Nikon).

### 4.7 DNA extraction

Each group of the squid mantle was placed in an oven at 60°C and dehydrated overnight. The next day, 1000 mg of the sample was weighed and transferred into tubes. First, 500 μL DNA extraction and protease K solution were added into each tube and digested in a 55°C water bath for 1 h. Then, an equal volume of 500 μL phenol/chloroform (1:1) mixture was added into tubes, mixed for 10 times, and the samples were centrifuged at 12,000 rpm for 10 min at 4°C. The supernatant was drained gently into the new tubes and phenol/chloroform/isoamyl alcohol solution (25:24:1 added in equal volumes) was added and centrifuged at 12,000 rpm for 10 min at 4°C. The supernatant was poured off carefully, 75% alcohol was added for precipitation, and centrifuged at 12,000 rpm for 5 min, at 4°C two times. The remaining alcohol in the EB tubes was volatilized. Then, the DNA content was measured using a spectrophotometer (ND‐ONE‐A1501015).

### 4.8 Cell isolation and culture

Human corneal epithelium cells (HCECs) were purchased from the American Type Culture Collection. Cells were resuscitated, centrifuged, and suspended in DMEM/Ham's F12 (Life Technologies) supplemented with 6% fetal bovine serum (Life Technologies), 7 ng mL^−1^ epidermal growth factor (Pep Rotech), 7 μg mL^−1^ insulin (Sigma‐Aldrich), and 25 μg mL^−1^ penicillin and 25 μg mL^−1^streptomycin (Life Technologies). The cells were then seeded on a plastic dish and incubated at 37°C, under 5% CO_2_ and 95% humidified air.

### 4.9 Cytotoxicity of extractable DSMS

Samples were washed with PBS solution and DDW, respectively. They were cut into a small piece with a 2.5‐mm trephine, and then immersed into sterile water with penicillin and streptomycin solution (PS) for 48 h for thorough cleaning. The liquid was changed every 2 h for the first 12 h, and then every 4 h for the next 12 h. The samples were drained on sterile filter paper and subjected in the ultraviolet clean bench for further sterilization under ultraviolet irradiation. HCECs were inoculated into 96‐well plates at a cell density of 10,000 cells per well, and pre‐cultured for 24 h. The culture medium was replaced with the above leaching liquor. Leaching liquor is a cell culture medium that has been incubated with the DSMS extract for 24 h at 37°C. At the time point of 24 and 48 h after changing medium, 10 μL CCK‐8 (Beyotime Biotechnology) solution was added into each well and incubated. The absorbance was detected at 450 nm by a Microplate Reader (Bio‐Tek Instruments).

### 4.10 Wound healing assay

For wound healing assays, the ibidi Culture‐Insert 2 Well (ibidi Gmbh) was placed in each well of 12‐well plate, and eight inserts were used for each treatment, each container separated by a 500‐μm wall.^51^ About 100 μL of HCE cell suspension was subjected to each of the Culture‐Insert 2 Wells, and the cells were incubated for 24 h at 37°C and 5% CO_2_. After waiting for the cells to fill the container, the Culture‐Insert 2 well was gently removed with sterile tweezers, followed by washing the cell layer two times with PBS to remove the cell debris and non‐attached cells, and then 700 μL of the soak solution was added. The samples were placed into tubes with DMEM/Ham's F12 medium and soaked overnight to prepare the leaching liquor. Photographs were taken under the microscope at 0, 12, and 24 h, and the healing area was calculated using ImageJ software.

### 4.11 Rat dorsal muscle implantation

All 24 male rats were purchased from the Experimental Animal Center of Xiamen University. Animals were selected according to a simple random method, and those with diseases were excluded before the experimental treatment. The protocol was approved by the Ethics Committee for Laboratory Animals of Xiamen University. Animal studies conformed to ARRIVE guidelines. The rats were divided into eight groups. To simulate the biological change of DSMS in the body, each group of DSMS was cut into 1 cm × 1 cm × 0.5 cm pieces. After the rats were anesthetized, the graft was implanted into the left back muscle of the rat, and the muscle and skin were sutured sequentially with 4‐0 cosmetic sutures. Samples of the peripheral tissues were separated and stained at 1, 2, and 4 weeks postoperatively. Then, the muscles were taken in turn, and frozen embedded for slicing.

### 4.12 Vibrating microtome

The samples for animal study were prepared as follows: The instrument was opened (LEICA VT 1200S) and the blade with a hexagonal screwdriver was secured. DDW was poured into the buffer tray and the buffer tray was snapped onto the dovetail slot. The tissue block was subjected to the sample holder and the height of the sample holder was adjusted to position the sample close to the slicing position. The slicing speed and thickness (350 μm) were set on the control board and the tissue blocks were sliced. The slices were picked up with a brush and placed in the box containing DDW.

### 4.13 Rabbit corneal interlaminar implantation

Eighteen male New Zealand White rabbits (2.5–3.0 kg) were selected for this study. In three normal controls, three rabbits underwent sham operation on the right eye (without implantation), six rabbits received the DSMS (thickness = 300 μm) implantation, and six rabbits received the DSMS (thickness = 350 μm) implantation. Each rabbit received the procedure only in the right eye. All rabbits were acclimatized to the laboratory conditions at the animal center of Xiamen University for 2 weeks before surgery. Animals were anesthetized with intra‐muscular injection of xylazine hydrochloride injection (0.1 mL/kg, Grand Animal Pharmaceutical) and 10% pentobarbital sodium (0.3 mL/kg). Before operation, procaine hydrochloride eye drops were used (Alcon Company). The anterior stroma was separated horizontally with a jeweler's knife until a circular capsule was formed with at least 6 mm in diameter. Separated 300 and 350 μm DSM (diameter of 5 mm) was implanted into the corneal pockets, and 10‐0 nylon suture (MAIN) was used to seal the periscleral incision.^32^, ^46^ Colistin drops and tobramycin dexamethasone drops (ALCON) were used to treat the surgical eyes, three times a day for 1 week after surgery. The sutures were removed at 1‐week follow‐up. The animals were euthanatized after the fourth and eighth weeks, respectively. The eyeballs were separated and fixed in 4% paraformaldehyde solution (Sigma‐Aldrich) for 1 week, and the corneas were peeled off and embedded in paraffin (Sigma‐Aldrich). The study protocol was approved by the Experimental Animal Ethics Committee of Xiamen University. Animals were used according to the Statement of the Society for Ophthalmic and Vision Research on the Use of Animals in Ophthalmic and Vision Research. Animal studies conformed to ARRIVE guidelines.

### 4.14 Slit lamp, AS‐OCT, SS‐OCTA, and confocal microscopy

Slit lamp was used to examine the graft attachment, corneal infection, and neovascularization on the day of operation and 1, 2, 4, and 8 weeks after operation. Anterior segment optical coherence tomography (AS‐OCT, RT‐100, Optovue Inc.) was done at 4 and 8 weeks after surgery. The average thickness of the central cornea was determined automatically by the built‐in OCT software. In the eighth week, swept‐source OCTA (SS‐OCTA, BM‐400K BMizar, TowardPi Medical Technology) was used for 3D view of the anterior segment and corneoscleral blood flow. The corneal epithelial cells, stromal cells, grafts, and endothelial cells were scanned using in vivo confocal microscope (Heidelberg Engineering Inc.). Carbomer Eye gel (Bausch & Lomb) was used as an immersion solution throughout the scan.

### 4.15 H&E and Masson staining of cornea

The rabbits were killed at 4 and 8 weeks after the operation and the eyeballs were fixed in 4% paraformaldehyde (Sigma‐Aldrich) for a week. Subsequently, the corneal tissue was embedded in paraffin and sliced. Samples were stained with H&E (Auragene) and Masson, and the slices were observed under a light microscope (Nikon).

### 4.16 Immunofluorescence staining

The steps for fluorescent staining are as follows: Paraffin sections were dewaxed with xylene and dehydrated with gradient alcohol concentrations. The sections underwent antigen repair in antigen repair solution by subjecting to microwave for 10 min and then cooled to RT. Subsequently, they were treated with a 0.2% Triton X‐100 solution for 20 min and washed in PBS three times for 5 min. Sections were then sealed with 2% BSA for 1 h. Subsequently, infiltrating macrophages and myofibroblasts in the cornea were stained with anti‐F480 antibody (Abcam) and anti‐α‐SMA (Abcam), respectively. Keratocytes were stained with aldh3a1 antibody (ProteinTech Group) and integrin β1 antibody (ab Clone) to assess the distribution of keratocyte at 4°C overnight. The next day, sections were washed with PBS three times for 10 min. Then sections were incubated in secondary antibody solution Alexa Fluor 488‐Conjugated IgG (1:200, Catalog number A11055, Invitrogen) for 1 h at RT, and then washed with PBS three times for 10 min. Finally, the sections were sealed with DAPI and observed by fluorescence microscope (LeicaDM2500).

### 4.17 Statistical analysis

All data were expressed as mean ± standard deviation (SD). According to the normality of the data distribution, using GraphPad Prism 9.0 software, the statistical significance was evaluated by a one‐way analysis of variance through Tukey's post hoc test and unpaired two‐tailed Student's *t* test. A *p* value less than 0.05 was considered to be statistically significant.

## 5 CONCLUSION

In conclusion, we constructed a novel tissue‐engineered corneal stroma material by decellularization and altering the transparency of the squid mantle. Subsequently, a series of in vitro and in vivo experiments confirmed that DSMS exhibited a suitable light transmittance, low immunogenicity, and good histocompatibility, which accorded with the basic characteristics of new tissue‐engineered corneal stroma materials. Our results indicate that the biomaterial from squid could be a potential marine source for corneal tissue engineering.

## AUTHOR CONTRIBUTIONS

**Honghua Kang:** Conceptualization (lead); data curation (lead); formal analysis (lead); investigation (lead); methodology (lead); supervision (lead); visualization (lead); writing – original draft (lead); writing – review and editing (lead). **Yi Han:** Conceptualization (lead); data curation (lead); investigation (lead); methodology (lead); supervision (lead); visualization (lead); writing – original draft (lead); writing – review and editing (lead). **Mengyi Jin:** Investigation (equal); methodology (equal); project administration (equal); visualization (equal); writing – original draft (equal). **Lan Zheng:** Data curation (equal); investigation (equal); methodology (equal). **Zhen Liu:** Investigation (equal); project administration (equal); supervision (equal). **Cheng Li:** Conceptualization (lead); funding acquisition (lead); project administration (lead); supervision (lead); writing – original draft (lead); writing – review and editing (lead).

## CONFLICT OF INTEREST STATEMENT

The authors declare no conflict of interest.

### PEER REVIEW

The peer review history for this article is available at https://www.webofscience.com/api/gateway/wos/peer-review/10.1002/btm2.10531.

## Supporting information


**DATA S1.** Supporting Information

## Data Availability

All data to support the findings from this study will be made available to interested investigators. The data sets used and/or analyzed during the current study are available from the corresponding author upon reasonable request.
