# Supplementary material for: Decellularized squid mantle scaffolds as tissue‐engineered corneal stroma for promoting corneal regeneration
Source: Bioeng Transl Med. 2023 May 9;8(4):e10531. doi: 10.1002/btm2.10531 (PMC10354768; doi:10.1002/btm2.10531)
Supplement: Supplementary file 1 — DATA S1. Supporting Information [file BTM2-8-e10531-s001.docx]

## Decellularized squid mantle scaffolds as tissue engineered corneal stroma for promoting corneal regeneration

## Author names and affiliations

Honghua Kang ^a, b, 1^, Yi Han ^a, b, 1^, Mengyi Jin ^a, b^, Lan Zheng ^a, b^, Zhen Liu ^a^, Yuhua Xue ^c *^, Zuguo Liu ^a, b, d *^, Cheng Li ^a, b, d *^

1. Eye Institute & Affiliated Xiamen Eye Center, School of Medicine, Xiamen University, Xiamen, 361102, China
2. Fujian Provincial Key Laboratory of Ophthalmology and Visual Science, School of Medicine, Xiamen University, Xiamen, 361102, China
3. School of Pharmaceutical Sciences, Xiamen University, Xiamen, 361102, China
4. Department of Ophthalmology, the First Affiliated Hospital of University of South China, Hengyang, Hunan, 421001, P. R. China

^1^ These authors contributed equally to this work.

**Supporting Information**

**Materials and Methods**

**Evaluation of *in vitro* biodegradation resistance**

Collagenase I was used for the *in vitro* enzyme degradation under accelerated conditions (37 °C) to evaluate the biodegradation resistance of the DSMS (125 U/mg, Sigma, USA)^1^. Initially, each group of samples were cut into slices with 6 mm diameter and 0.1 cm thickness and incubated in 2 mL PBS solution (collagenase concentration in PBS 20 U/mL) at 37 °C for 7 days. The samples were checked for any change in size over time to assess the resistance of DSMS against enzymatic degradation. The solution was changed every 3 days for the ease of observation and to maintain an adequate collagenase activity.

**Determination of Residual SDS Content**

The protocol and Residual SDS Content Kit were provided from Sangon Biotech (Shanghai) Co., Ltd. Standard curve established as follow, eight groups of samples were diluted in appropriate multiples, then 50 μL of sample dilution was added, and another tube was added with 50 μL of distilled water as a blank control. Then add 50 μL of solution A to each tube and mix well. Then add 1.5 mL of Solution B to each tube, shake vigorously and mix thoroughly for 3 min for extraction. Centrifuge at 5000 rpm for 5 min at room temperature, and take the upper layer of liquid on the spectrophotometer to measure the OD value of each tube of sample solution at A499. Calculate the average of the OD values of the two sample dilutions at A499. Determine the SDS concentration of the sample dilutions to be tested on the standard curve based on the average of the two sample dilutions at A499.

**Cell live/dead staining**

Human corneal epithelium cells (HCECs) were seeded in 12-well plates at a concentration of 1x 10 ^4^ cells/well and allowed to adhere overnight. After the cells were completely adhered, the culture solution was aspirated and washed once with phosphate-buffered saline (PBS). Subsequently, 500 ul of Calcein AM/PI assay working solution (Beyotime Biotechnology, Shanghai, China) was added to each well and incubated at 37 °C for 30 min in the dark. After incubation, the cells were observed under a fluorescence microscope (Calcein AM: green fluorescence, Ex/Em=494/517nm. PI: red fluorescence, Ex/Em=535/617nm).

**Immunofluorescence staining**

HCE was fixed in 4% paraformaldehyde (PFA) for 20 min, and the cells were washed three times with PBS, followed by incubation with 0.2% Triton X 100 for 20 min. After three washes with PBS (5 min each), the samples were incubated with 2% bovine serum albumin (BSA) for 1 h at room temperature. Subsequently, the cells were incubated with the primary antibody ZO-1 (1: expression vector, Carlsbad, Calcium, USA), overnight at 4°C, and washed three times with PBS for 10 min each. The cells were then incubated with the secondary antibody Alexa Fluor 488 (donkey anti-mouse) and washed with PBS for 10 min each. The cells were re-stained with DAPI and then viewed under a laser scanning confocal microscope (LeicaDM2500).

## References

1. Hong H, Huh M Il, Park SM, Lee KP, Kim HK, Kim DS. Decellularized corneal lenticule embedded compressed collagen: Toward a suturable collagenous construct for limbal reconstruction. *Biofabrication*. 2018;10(4). doi:10.1088/1758-5090/aad1a4

**Table 1:** Transparency criteria for cornea during post-operation observation

|  | **Grade 0** | **Grade 1** | **Grade 2** | **Grade 3** | **Grade 4** |
| --- | --- | --- | --- | --- | --- |
| **Area of corneal opacity** | 0 | 1%–25% | 26%–50% | 51%–75% | 76%–100% |
| **Density of corneal opacity** | Not cloudy | Slight cloudiness, pupil visible | Cloudy, mostly pupil discernible | Moderate cloudiness, partial pupil discernible | Cloudy, opacity, pupil invisible |


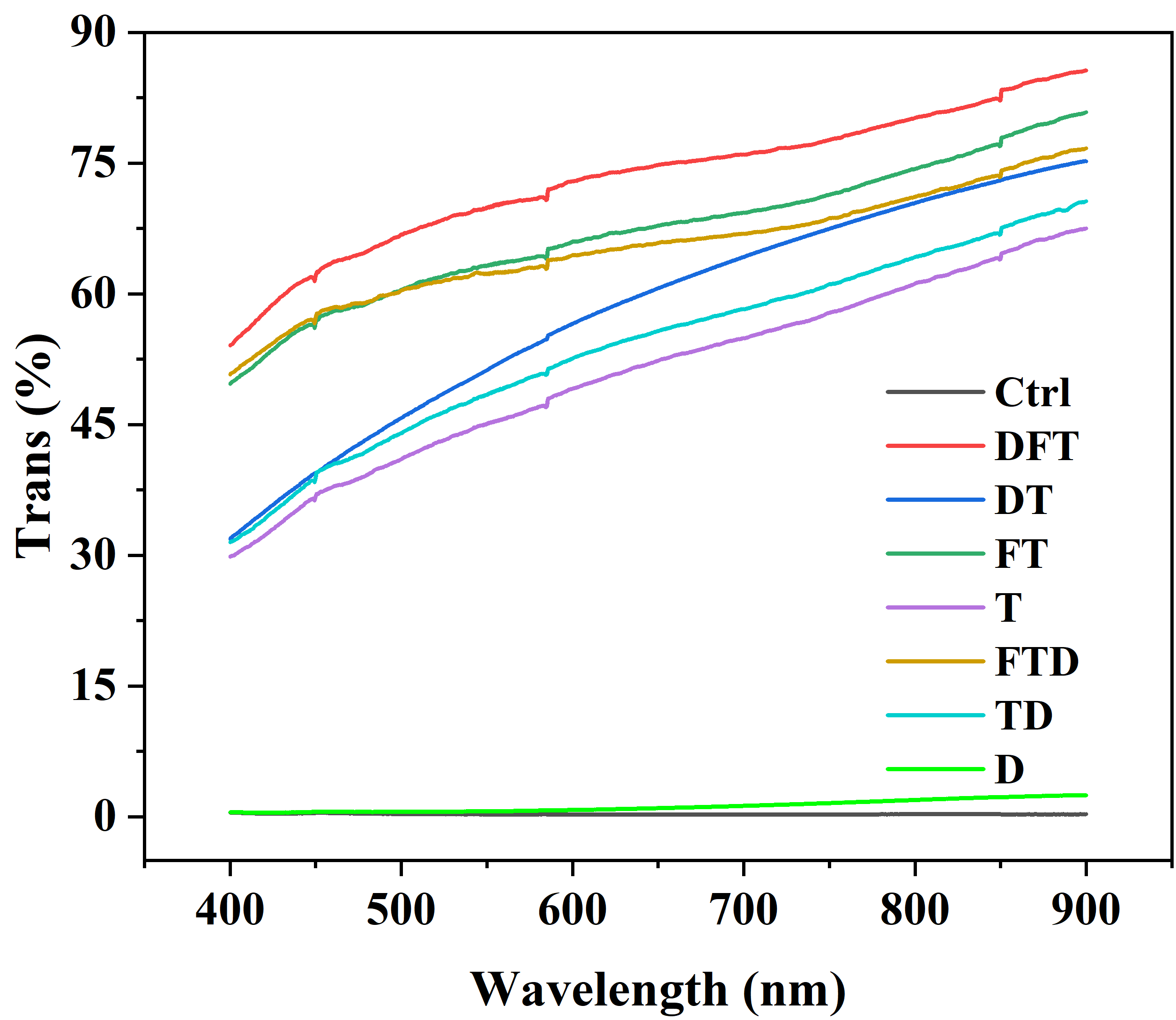


**Figure S1.** The spectral transmission curve of different material. Untreated group (Ctrl), decellularized and fixed and transparent group (DFT), decellularized and transparent group (DT), fixed and transparent group (FT), transparent group (T), transparent-fixed-decellularized group (TFD), transparent and decellularized group (TD) and decellularized group (D)


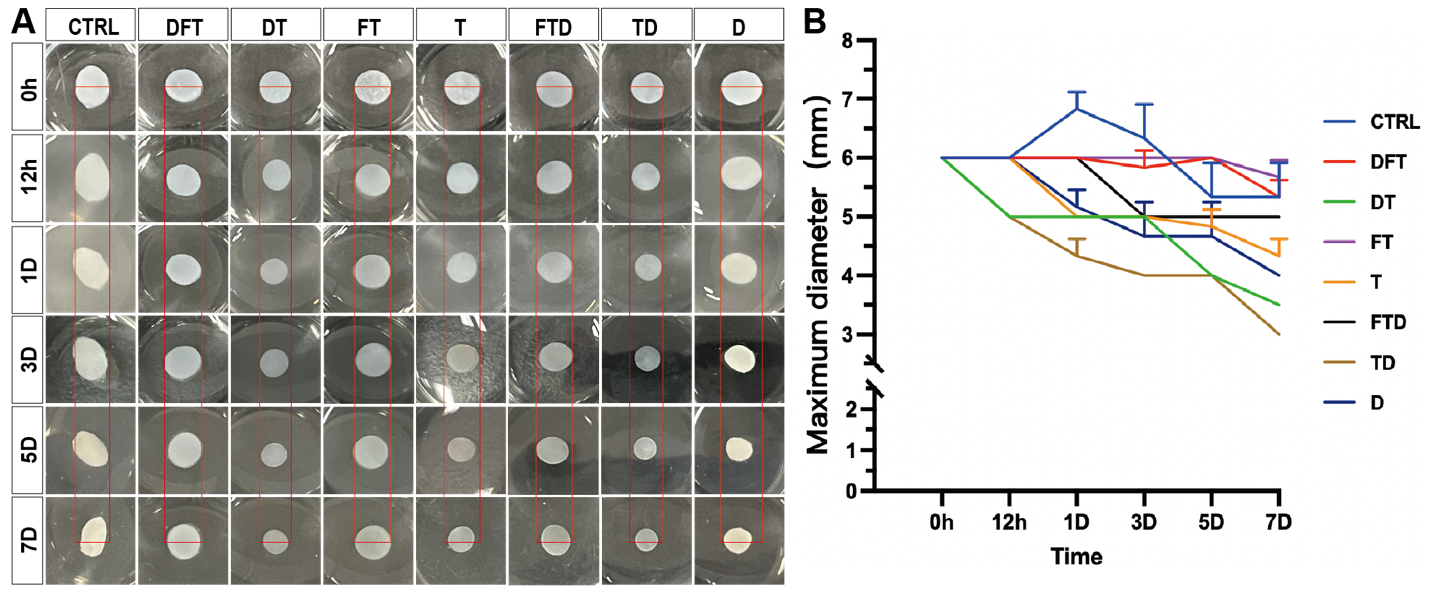


**Figure S2.** (A) Structural changes of DSMS against *in vitro* enzymatic degradation at different time intervals. (B) The graph of enzymatic degradation. Untreated group (Ctrl), decellularized and fixed and transparent group (DFT), decellularized and transparent group (DT), fixed and transparent group (FT), transparent group (T), transparent-fixed-decellularized group (TFD), transparent and decellularized group (TD) and decellularized group (D)


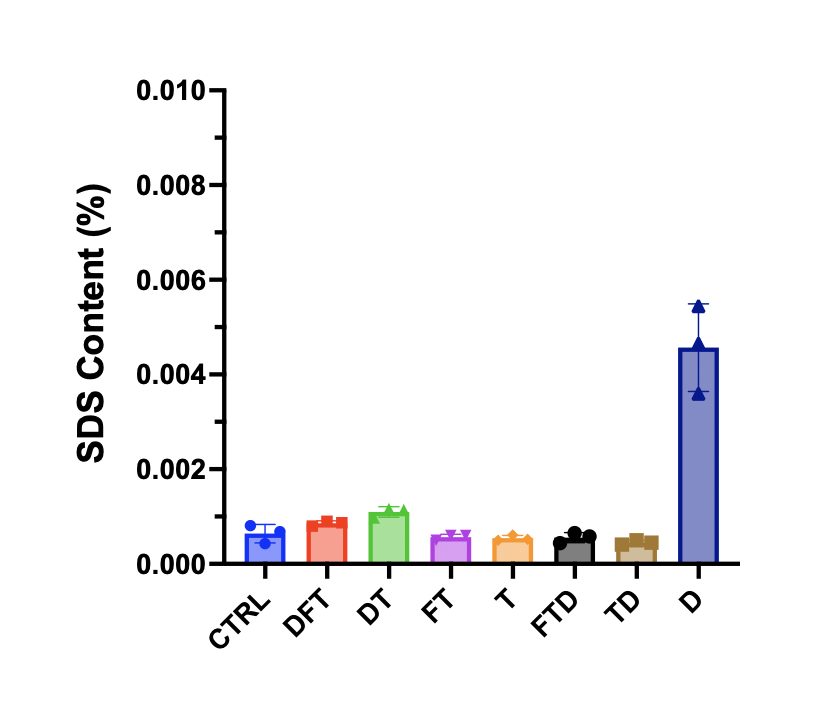


**Figure S3.** The residual SDS content in the materials. Untreated group (Ctrl), decellularized and fixed and transparent group (DFT), decellularized and transparent group (DT), fixed and transparent group (FT), transparent group (T), transparent-fixed-decellularized group (TFD), transparent and decellularized group (TD) and decellularized group (D)


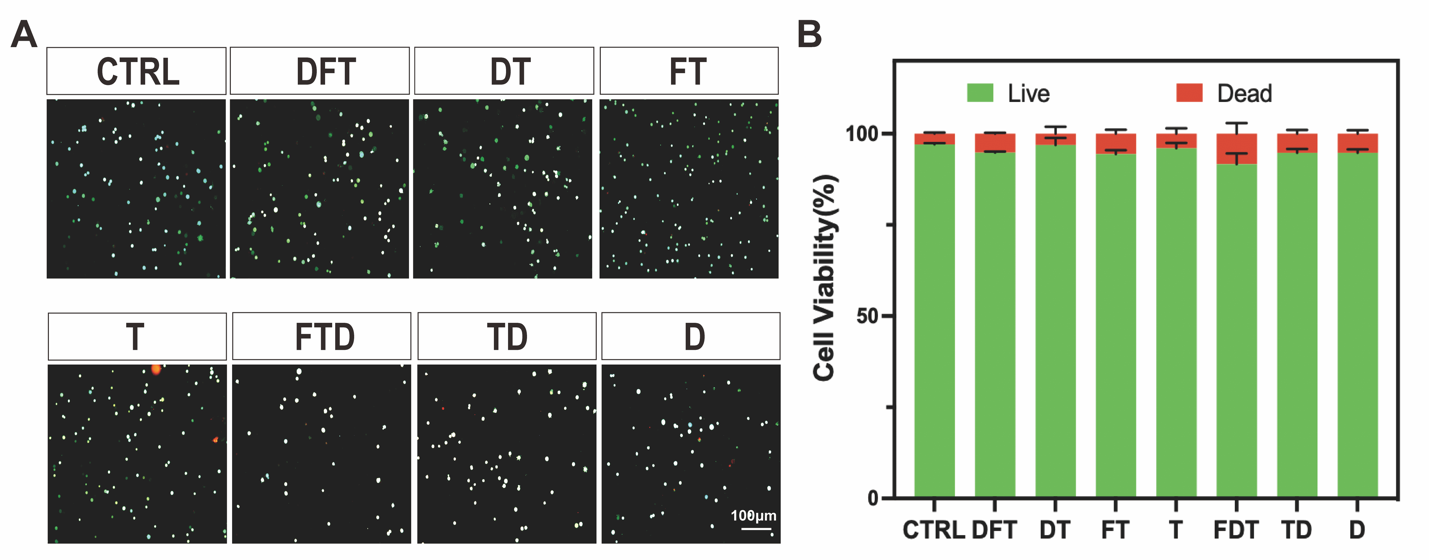


**Figure S4.** (A) Viability of corneal epithelial cells as evaluated in a Live/Dead assay (viable cells in green, PI-positive dead cells in red); (B) Quantification of Live/Dead assay data shown as a graph. Untreated group (Ctrl), decellularized and fixed and transparent group (DFT), decellularized and transparent group (DT), fixed and transparent group (FT), transparent group (T), transparent-fixed-decellularized group (TFD), transparent and decellularized group (TD) and decellularized group (D)


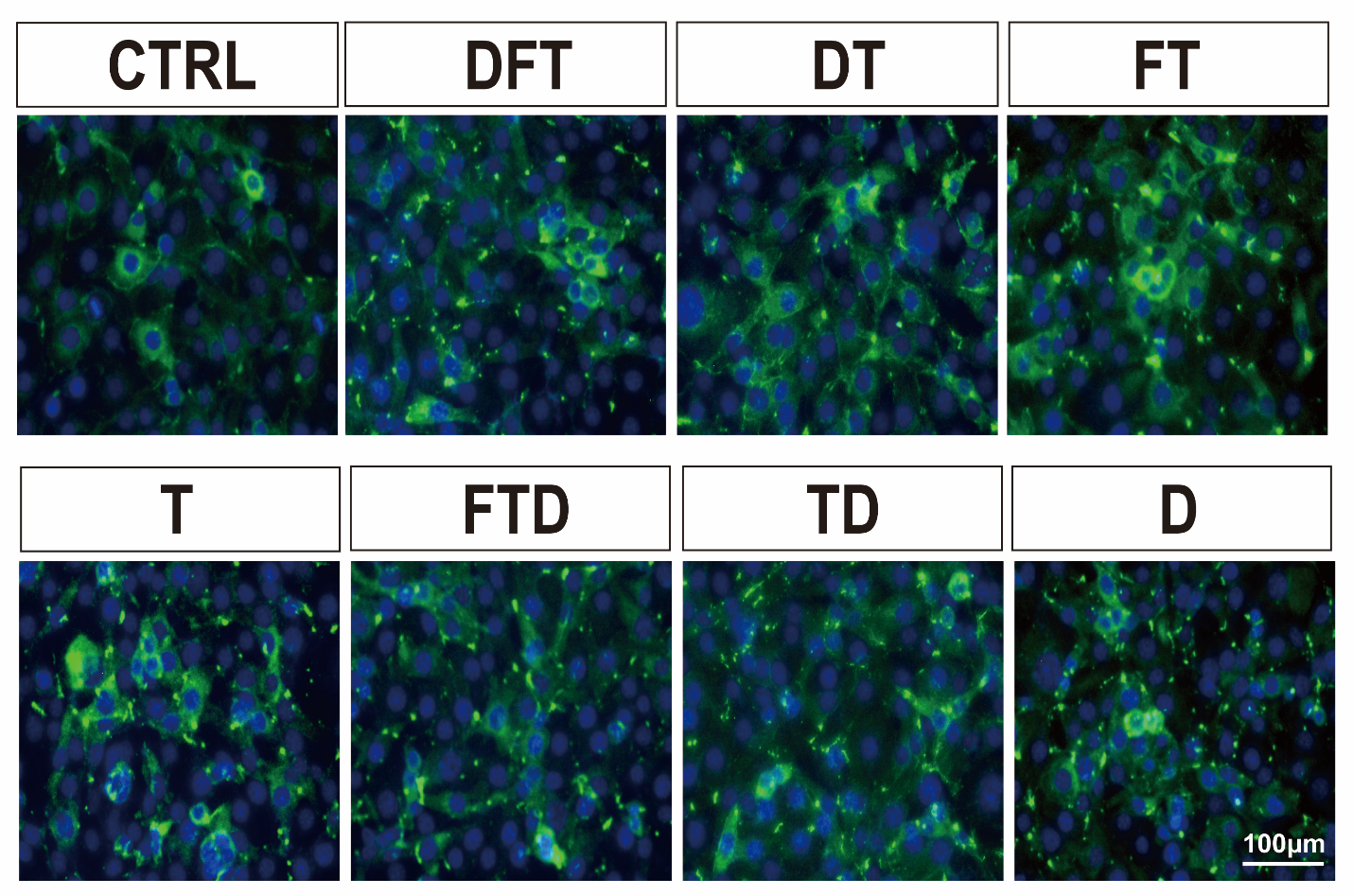


**Figure S5.** Immunofluorescence staining with ZO-1 of corneal epithelial cells. Untreated group (Ctrl), decellularized and fixed and transparent group (DFT), decellularized and transparent group (DT), fixed and transparent group (FT), transparent group (T), transparent-fixed-decellularized group (TFD), transparent and decellularized group (TD) and decellularized group (D).
